# Stroke and Bleeding Risks With Non–Vitamin K Oral Anticoagulants in Nonvalvular Atrial Fibrillation

**DOI:** 10.1001/jamanetworkopen.2026.9082

**Published:** 2026-04-24

**Authors:** Marie C. Bradley, Andrew L. Simon, Joy Kolonoski, David J. Graham, Rongmei Zhang, John G. Connolly

**Affiliations:** 1 Office of Medical Policy, Center for Drug Evaluation and Research, US Food and Drug Administration, Silver Spring, Maryland; 2 Department of Population Medicine, Harvard Pilgrim Health Care Institute, Boston, Massachusetts; 3 Office of Surveillance and Epidemiology, Center for Drug Evaluation and Research, US Food and Drug Administration, Silver Spring, Maryland; 4 Office of Biostatistics, Center for Drug Evaluation and Research, US Food and Drug Administration, Silver Spring, Maryland

## Abstract

**Importance:**

A study using Medicare data concluded that among patients aged 65 years or older, rivaroxaban had a less favorable benefit-harm profile compared with other non–vitamin K oral anticoagulants (NOACs); however, it is unclear whether this finding persists in younger users.

**Objective:**

To compare major extracranial bleeding (MEB), gastrointestinal bleeding (GIB), intracranial hemorrhage (ICH), and thromboembolic stroke in individuals aged younger than 65 years using non–vitamin K oral anticoagulants (NOAC users) for nonvalvular atrial fibrillation (NVAF).

**Design, Setting, and Participants:**

This cohort study using health care claims data between October 2010 and February 2022 in the FDA Sentinel System included standard dose NOAC (rivaroxaban, apixaban, and dabigatran) users with NVAF aged 21 to 64 years. Analyses conducted between December 2022 and August 2023.

**Main Outcomes and Measures:**

Hazard ratios (HRs) and 95% CIs were estimated for each outcome (MEB, GIB, ICH, and thromboembolic stroke) in inverse probability of treatment-weighted pairwise comparisons: rivaroxaban vs apixaban, rivaroxaban vs dabigatran, and dabigatran vs apixaban.

**Results:**

A total of more than 173 000 patients (mean [SD] age, 56.6 [7.23] years; 27.5% female; 72.5% male) were included across the 3 exposure cohorts. The number of NOAC users for each pairwise comparison were rivaroxaban (57 932) vs apixaban (96 057), rivaroxaban (57 399) vs dabigatran (20 188), and dabigatran (20 163) vs apixaban (96 668). Rivaroxaban was associated with higher risks of MEB and GIB compared with apixaban (MEB: HR, 1.91; 95% CI, 1.56-2.34; GIB: HR, 1.92, 95% CI, 1.54-2.39), while differences in thromboembolic stroke prevention were not significant (HR, 1.05; 95% CI, 0.77-1.44). Dabigatran was associated with higher thromboembolic stroke risk (rivaroxaban vs dabigatran: HR, 0.61; 95% CI, 0.39-0.94; dabigatran vs apixaban: HR, 1.74; 95% CI, 1.13-2.68).

**Conclusions and Relevance:**

These findings suggest that for patients aged younger than 65 years treated with NOACs for NVAF, apixaban was associated with the most favorable benefit-harm profile. While the results suggest that rivaroxaban may have provided greater stroke prevention than dabigatran, it was associated with higher bleeding risks than apixaban without additional stroke prevention. The higher thromboembolic stroke risks observed with dabigatran in younger patients suggest important age-related differences in effectiveness that warrant further investigation.

## Key Points

**Question:**

Does rivaroxaban have a less favorable benefit-harm profile compared with other non–vitamin K oral anticoagulants (NOACs) in patients younger than 65 years treated for atrial fibrillation?

**Findings:**

In this cohort study of more than 173 000 patients with nonvalvular atrial fibrillation across 3 exposure cohorts, rivaroxaban use was associated with an increased risk of major extracranial and gastrointestinal bleeding, as well as a marginally increased risk of intracranial hemorrhage compared with apixaban. Thromboembolic stroke risk was similar in apixaban and rivaroxaban users, but higher risks were seen in dabigratan users compared with both.

**Meaning:**

These findings suggest, for NOAC users younger than 65 years, rivaroxaban may provide greater protection against thromboembolic stroke than dabigatran but is less favorable than apixaban due to increased bleeding risks.

## Teaser

This cohort study compares the relative risks and benefits of non–vitamin K oral anticoagulants in patients younger than 65 years of age with nonvalvular atrial fibrillation.

## Introduction

Non-vitamin K oral anticoagulants (NOACs) are increasingly used for multiple indications, including stroke prevention in patients with nonvalvular atrial fibrillation (NVAF). Randomized clinical trials have demonstrated the effectiveness of NOACs in preventing stroke in NVAF and reducing intracranial bleeding compared with warfarin.^1,2,3,4^ However, no randomized clinical trials have compared NOACs with each other in terms of their bleeding risks and effectiveness in preventing stroke in patients with NVAF. Several observational studies^5,6,7,8,9,10,11,12,13,14,15,16^ conducted head-to-head comparisons of NOAC safety and effectiveness, but few included substantial numbers of individuals using NOACS (NOAC users) younger than 65 years of age, and many were limited by inadequate adjustment of confounding, inappropriate outcome ascertainment, small study sizes, and prevalent user designs. A large well-designed study^17^ using Medicare claims data designed to overcome these limitations concluded that rivaroxaban had a less favorable benefit-harm profile compared with other NOACs among patients aged 65 years or older with NVAF. Nonetheless, it remains unclear whether a similar benefit-harm profile persists in younger users. This cohort study aimed to examine the comparative safety and effectiveness of the most commonly used NOACs in the US among those younger than 65 years with NVAF in the Food and Drug Administration (FDA) Sentinel System. The FDA Sentinel System is an active-surveillance system that uses routinely collected healthcare data from a distributed network of databases (eg, health care claims and electronic health records) to monitor the safety of FDA-regulated products. It enables the FDA to rapidly evaluate potential safety signals and conduct large-scale postmarketing studies to inform regulatory decision-making and protect public health.^18^

## Methods

This cohort study was conducted in the FDA Sentinel System as a public health surveillance activity under the authority of the FDA, and institutional review board oversight was not obtained consistent with 45 CFR §46.102(l)(2).^19,20^ Deidentified administrative health care claims data that were routinely collected and maintained by Sentinel data partners were used in the analysis. Accordingly, informed consent was not required. This study is reported in accordance with the Strengthening the Reporting of Observational Studies in Epidemiology (STROBE) reporting guideline for cohort studies. Data were analyzed between December 2022 and August 2023.

### Study Design and Data Source

This cohort study was conducted using data from 5 data partners that contribute to the FDA Sentinel System, including 4 national commercial insurance plans and 1 state Medicaid partner. The FDA Sentinel System includes health care claims data from medical encounters (inpatient and outpatient diagnoses and procedures) and outpatient pharmacy (retail and mail order filled prescriptions) accrued during health plan enrollment periods. These data are routinely quality checked and transformed into the Sentinel Common Data Model.^18^

### Study Population

Standard dose NOAC initiators aged 21 to 64 years between October 19, 2010, and February 28, 2022, were included in the study. Patients were eligible for study inclusion if on the date of a qualifying NOAC dispensing (index date) they had at least 6 months of prior continuous medical and drug coverage, were aged 21 to 64 years, and during the preceding 6 months had an inpatient or outpatient diagnosis of NVAF or for nonvalvular atrial flutter based on *International Classification of Diseases, Ninth Revision, Clinical Modification (ICD-9-CM)* and *International Statistical Classification of Diseases, Tenth Revision, Clinical Modification (ICD-10-CM)* coding.

Patients were excluded if in the 6 months preceding the index date they had evidence of anticoagulant use (including edoxaban or warfarin), dialysis, a kidney replacement, a potential alternative indication for anticoagulation (deep vein thrombosis, pulmonary embolism, joint replacement, mitral stenosis, heart valve replacement or repair), or had an institutional stay encounter (index date only).

### Exposure

All NOACs available in the US at a standard dose for NVAF were considered to define index dates (Figure). Standard doses were defined as dabigatran 150 mg twice daily, rivaroxaban 20 mg once daily, and apixaban 5 mg twice daily. We did not require dispensing subsequent to the index-defining dispensing to be at the standard dose. Edoxaban was not included due to low use during the study period. Three pairwise NOAC-NOAC comparisons were conducted: rivaroxaban vs dabigatran, rivaroxaban vs apixaban, and dabigatran vs apixaban.

**Figure.**
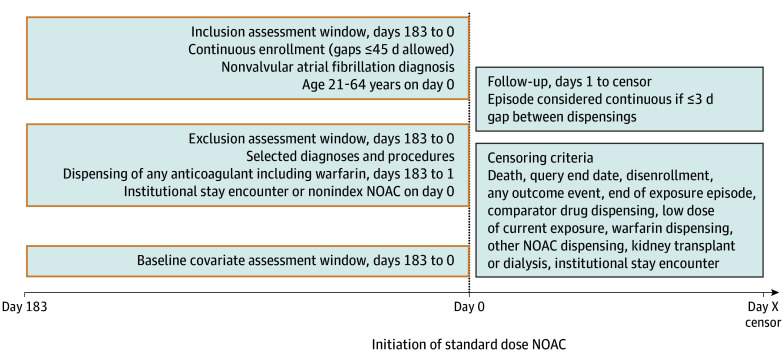
Diagram of Study Design NOAC indicates non–vitamin K oral anticoagulant.

### Outcomes and Follow-Up

The outcomes were an inpatient principal diagnosis for major extracranial bleeding (MEB), which included gastrointestinal bleeding (GIB), GIB only, intracranial hemorrhage (ICH), and thromboembolic stroke. Thromboembolic stroke was defined using a validated algorithm with a positive predictive value (PPV) of 88% to 95%.^21,22,23^ ICH, MEB, and GIB were defined using a modified version of a validated algorithm for hospitalized bleeding by Cunningham et al^24^ (PPV 89%-97%). Modifications included the addition of codes for ICH accompanied by nonpenetrating head trauma to capture events that might have been preceded by a fall, and removing the requirement for major bleeding to have been treated with red blood cell or whole blood transfusion when involving a critical site (ie, intraarticular, pericardial, retroperitoneal)^24^ or when resulting in death. These *ICD-9-CM*–based algorithms were mapped to the *ICD-10-CM* coding system via forward-backward mapping^25,26^ using the 2017 General Equivalence Mappings.^27^

Follow-up began on the day after the first dispensing of a study NOAC and continued until the first occurrence of any of the following: (1) outcome occurrence, (2) switching to a different anticoagulant, (3) disenrollment, (4) recorded death, (5) end of exposure episode, or (6) end of query period (February 28, 2022) or end of available data at the data partner. Exposure episodes were considered continuous if gaps in days supplied were 3 days or less. Three days was chosen as a gap allowance based on the half-life of NOACs and the requirement that patients had to keep receiving NOACs during follow-up. An extension of 3 days was added to the end of each exposure episode.

### Covariates

During the 6 months preceding cohort entry, information on demographic characteristics, chronic medical conditions, risk factors for cardiovascular and bleeding events (including CHA2DS2-VASc [congestive heart failure, hypertension, age ≥75 years, diabetes, prior stroke or transient ischemic attack, or thromboembolism, vascular disease, age 65-74 years, sex category] and HASBLED [hypertension, abnormal kidney and liver function, stroke, bleeding, labile international normalized ratio, elderly, drugs or alcohol] scores),^28,29^ and health care use were collected for each patient, as were data on dispensed medications. CHA2DS2-VASc and HASBLED scores were entered into propensity score (PS) models as composite categorical variables rather than as individual score components. Due to our cohort restriction to patients aged younger than 65 years, the age components of both scores contributed 0 points for all patients.

### Statistical Analysis

To adjust for potential confounding, inverse probability of treatment weighting with stabilized average treatment effect weights were applied separately for each pairwise comparison: rivaroxaban vs apixaban, rivaroxaban vs dabigatran, and dabigatran vs apixaban. Logistic PS models predicting the likelihood of exposure were estimated using the described covariates, except for race and calendar year (due to completeness of capture and different market entry years), and were then used to estimate stabilized inverse probability weights targeting the average treatment effect. Patients in regions of PS nonoverlap were excluded from the analysis.^30^ The PS distributions and covariate balance between groups were examined before and after PS weighting using standardized mean differences, with absolute values greater than 0.10 indicating imbalance. To address extreme inverse probability weights, weight truncation was applied prior to generating effect estimates^31^ using 3 candidate weight truncation percentile thresholds: 0% (no truncation), 1%, and 2.5%.

After a review of the distribution of inverse probability treatment weights for the 3 truncation thresholds, blinded to effect estimates, the most appropriate threshold was selected and applied to all analyses for each pairwise comparison. For the rivaroxaban vs apixaban comparison, weight truncation was not applied. For the rivaroxaban vs dabigatran comparison, we truncated at the 2.5th and 97.5th percentile. For the dabigatran vs apixaban comparison, we truncated at the 1st and 99th percentiles. Cox proportional hazards regression was used to estimate the hazard ratios (HRs) and robust 95% CIs for each outcome (thromboembolic stroke, MEB, GIB, and ICH) for each pairwise comparison.^32^ Prespecified subgroup analyses were performed for all outcomes in categories defined by age, sex, CHA2DS2-VASc score, and HASBLED score. The PS was reestimated among patients in each subgroup category, and the same weight truncation level was applied as in the overall analysis, except for the CHAD2DS2-VASc and HASBLED subgroups in the dabigatran vs apixaban comparison, which required additional truncation at the 2.5th and 97.5th percentiles to avoid extreme weights.

Three separate pairwise comparisons were used as the modular nature of the Sentinel System tools precluded the use of a single multinomial PS model, which was used in a previous study.^17^ To assess the impact of this we replicated the findings from Graham et al^17^ using our proposed pairwise comparison approach within the Sentinel System’s Medicare data partner. The same study design and parameters as the original study were applied, which confirmed that the pairwise comparison approach, at least in NOAC users 65 years of age or older, did not materially alter the findings, supporting the decision to proceed with this method in the Sentinel System (eTables 1-3 in Supplement 1). Each pairwise comparison was a distinct Sentinel System analysis and therefore executed at a different calendar date between December 2022 and August 2023. Statistical significance was assessed using 2-sided 95% CIs. Estimates were considered statistically significant when the 95% CI interval did not include the null value (ie, a hazard ratio of 1.0) with an implied significance level of α = .05. Data were analyzed with SAS version 9.4 (SAS Institute).

## Results

More than 173 000 patients (mean [SD] age, 56.6 (7.23) years; 27.5% female; 72.5% male) were included. The analytic cohorts for each comparison (rivaroxaban vs dabigatran, rivaroxaban vs apixaban, and dabigatran vs apixaban) were constructed separately, and therefore there are minor differences in exposure cohorts between comparisons. New users were identified for each pairwise NOAC comparison: rivaroxaban (57 932) vs apixaban (96 057), rivaroxaban (57 399) vs dabigatran (20 188), and dabigatran (20 163) vs apixaban (96 668). Apixaban was the most widely used NOAC during the study period. Weighted baseline demographic and clinical characteristics for each pairwise comparison are shown in Table 1 and eTable 4 in Supplement 1. Across all cohorts, median follow-up was 62 (IQR, 32-126) days. Prior to weighting, there were minor differences in certain covariates that varied across comparisons. Comparing rivaroxaban with apixaban, rivaroxaban users had slightly lower mean (SD) CHA2DS2-VASc (1.6 [1.2] vs 1.8 [1.3]) and HASBLED (0.9 [0.7] vs 1.1 [0.8]) scores and were less likely to have acute kidney failure (4.6% vs 8.3%) or chronic kidney failure (4.6% vs 8.3%) (eTable 5 in Supplement 1). Comparing rivaroxaban with dabigatran, rivaroxaban users were more likely to have evidence of obesity (33.1% vs 26.1%) (eTable 6 in Supplement 1). Comparing dabigatran with apixaban, dabigatran users were more likely to be male (76.7% vs 70.6%), had lower mean (SD) CHA2DS2-VASc (1.5 [1.1] vs 1.8 [1.3]) and HASBLED (0.9 [0.7] vs 1.1 [0.8]) scores, and were less likely to have evidence of obesity (22.6% vs 40.0%) (eTable 7 in Supplement 1). After inverse probability of treatment weights were applied, cohorts were closely balanced for all covariates, with standardized mean differences less than 0.1.

**Table 1.** Weighted Select Baseline Characteristics of NOAC Users for Each Pairwise NOAC Comparison

Characteristic^a^	No. (%)					
	Rivaroxaban vs apixaban		Rivaroxaban vs dabigatran		Dabigatran vs apixaban	
	Rivaroxaban users	Apixaban users	Rivaroxaban users	Dabigatran users	Dabigatran users	Apixaban users
Patients, weighted No.	57 965	96 013	57 127	19 679	18 882	96 132
Age, mean (SD), y	56.6 (7.2)	56.7 (7.3)	56.3 (7.4)	56.2 (7.4)	56.6 (7.1)	56.8 (7.2)
Age, y						
21-49	9065 (15.6)	15 267 (15.9)	9684 (17.0)	3446 (17.5)	3031 (16.1)	14 792 (15.4)
50-64	48 900 (84.4)	80 746 (84.1)	47 443 (83.0)	16 233 (82.5)	15 851 (83.9)	81 340 (84.6)
Sex^b^						
Female	16 208 (28.8)	26 985 (28.1)	14 310 (25.0)	4821 (24.5)	5190 (27.5)	27 288 (28.4)
Male	41 756 (72.0)	69 027 (71.9)	42 817 (75.0)	14 858 (75.5)	13 692 (72.5)	68 845 (71.6)
Year						
2010	0	0	0	430 (2.2)	375 (2.0)	0
2011	44 (0.1)	0	56 (0.1)	6251 (31.8)	5640 (29.9)	0
2012	2495 (4.3)	0	2961 (5.2)	4391 (22.3)	4061 (21.5)	0
2013	5502 (9.5)	1512 (1.6)	6074 (10.6)	2326 (11.8)	2193 (11.6)	1511 (1.6)
2014	7059 (12.2)	4042 (4.2)	7547 (13.2)	1519 (7.7)	1478 (7.8)	4057 (4.2)
2015	6498 (11.2)	7189 (7.5)	6747 (11.8)	1075 (5.5)	1068 (5.7)	7248 (7.5)
2016	6428 (11.1)	9631 (10.0)	6369 (11.1)	1396 (7.1)	1493 (7.9)	9693 (10.1)
2017	6838 (11.8)	11 530 (12.0)	6486 (11.4)	955 (4.9)	1056 (5.6)	11 558 (12.0)
2018	6529 (11.3)	13 089 (13.6)	6106 (10.7)	642 (3.3)	725 (3.8)	13 092 (13.6)
2019	5742 (9.9)	14 758 (15.4)	5315 (9.3)	355 (1.8)	391 (2.1)	14 770 (15.4)
2020	4810 (8.3)	15 070 (15.7)	4398 (7.7)	200 (1.0)	226 (1.2)	15 062 (15.7)
2021	5129 (8.8)	16 992 (17.7)	4696 (8.2)	131 (0.7)	146 (0.8)	16 955 (17.6)
2022	890 (1.5)	2202 (2.3)	374 (0.7)	6 (0.0)	29 (0.2)	2185 (2.3)
CHA2DS2-VASc, mean (SD)	1.7 (1.2)	1.7 (1.2)	1.5 (1.2)	1.5 (1.1)	1.6 (1.2)	1.8 (1.2)
HASBLED, mean (SD)	1.0 (0.8)	1.0 (0.8)	0.9 (0.7)	0.9 (0.7)	1.0 (0.8)	1.0 (0.8)
Health characteristics						
Diabetes	16 109 (27.8)	27 271 (28.4)	14 772 (25.9)	4945 (25.1)	4936 (26.1)	27 609 (28.7)
Hypercholesterolemia	15 558 (26.8)	26 023 (27.1)	14 807 (25.9)	5084 (25.8)	4968 (26.3)	26 543 (27.6)
Hypertension	41 964 (72.4)	69 967 (72.9)	39 861 (69.8)	13 587 (69.0)	13 450 (71.2)	70 338 (73.2)
Kidney failure						
Chronic	3926 (6.8)	6726 (7.0)	2519 (4.4)	827 (4.2)	1122 (5.9)	7063 (7.3)
Acute	4452 (7.7)	7529 (7.8)	2511 (4.4)	775 (3.9)	1122 (5.9)	7923 (8.2)
Obesity	21 546 (37.2)	36 056 (37.6)	17 334 (30.3)	5598 (28.4)	6389 (33.8)	35 462 (36.9)
Peptic ulcer disease	198 (0.3)	341 (0.4)	139 (0.2)	45 (0.2)	63 (0.3)	375 (0.4)
Nicotine dependency	15 227 (26.3)	25 624 (26.7)	12 144 (21.3)	3854 (19.6)	4401 (23.3)	25 342 (26.4)
Hospitalized AMI						
Past 0-30 d	1281 (2.2)	2217 (2.3)	834 (1.5)	259 (1.3)	333 (1.8)	2323 (2.4)
Past 31-183 d	677 (1.2)	1132 (1.2)	395 (0.7)	115 (0.6)	165 (0.9)	1160 (1.2)
Coronary revascularization	4916 (8.5)	8362 (8.7)	3767 (6.6)	1259 (6.4)	1476 (7.8)	8760 (9.1)
Hospitalized heart failure	9004 (15.5)	15 287 (15.9)	7013 (12.3)	2297 (11.7)	2663 (14.1)	15 835 (16.5)
Outpatient heart failure	8641 (14.9)	14 775 (15.4)	7263 (12.7)	2399 (12.2)	2613 (13.8)	15 101 (15.7)
Other ischemic heart disease	15 777 (27.2)	26 600 (27.7)	14 367 (25.1)	4911 (25.0)	5031 (26.6)	27 202 (28.3)
Hospitalized stroke						
Past 0-30 d	737 (1.3)	1246 (1.3)	581 (1.0)	216 (1.1)	279 (1.5)	1388 (1.4)
Past 31-183 d	447 (0.8)	724 (0.8)	292 (0.5)	104 (0.5)	157 (0.8)	811 (0.8)
Transient ischemic attack	1843 (3.2)	3051 (3.2)	1710 (3.0)	611 (3.1)	646 (3.4)	3215 (3.3)
Other medical conditions (falls, fractures, syncope)	15 037 (25.9)	25 052 (26.1)	14 295 (25.0)	4925 (25.0)	1650 (8.7)	9006 (9.4)
Cardioversion	9265 (16.0)	15 254 (15.9)	9359 (16.4)	3255 (16.5)	3041 (16.1)	15 347 (16.0)
Cardioablation	2583 (4.5)	4261 (4.4)	2897 (5.1)	1027 (5.2)	907 (4.8)	4546 (4.7)
Hospitalized for bleeding	256 (0.4)	419 (0.4)	181 (0.3)	64 (0.3)	84 (0.4)	474 (0.5)
Medication use						
Estrogen replacement	1394 (2.4)	2294 (2.4)	1142 (2.0)	393 (2.0)	463 (2.5)	2354 (2.4)
H2-antagonist	1500 (2.6)	2579 (2.7)	1289 (2.3)	383 (1.9)	421 (2.2)	2635 (2.7)
NSAIDs	9324 (16.1)	15 575 (16.2)	8840 (15.5)	3004 (15.8)	2980 (15.8)	15 596 (16.2)
Proton pump inhibitors	11 829 (20.4)	19 994 (20.8)	10 614 (18.6)	3574 (18.2)	3750 (19.9)	20 250 (21.1)
SSRI antidepressants	7023 (12.1)	11 855 (12.3)	6320 (11.1)	2109 (10.7)	2201 (11.7)	11 923 (12.4)
Insulin	4128 (7.1)	7119 (7.4)	3498 (6.1)	1159 (5.9)	1230 (6.5)	7349 (7.6)
Metformin (biguanide)	9840 (17.0)	16 572 (17.3)	8942 (15.7)	2932 (14.9)	2925 (15.5)	16 542 (17.2)
Sulfonylureas	3031 (5.2)	5184 (5.4)	3518 (6.2)	1198 (6.1)	993 (5.3)	5315 (5.5)
Other diabetes medications	4715 (8.1)	7954 (8.3)	3994 (7.0)	1326 (6.7)	1408 (7.5)	8147 (8.5)
ACEI/ARB	29 029 (50.1)	48 349 (50.4)	27 630 (48.4)	9399 (47.8)	9308 (49.3)	48 563 (50.5)
Antiarrhythmics	16 491 (28.5)	27 333 (28.5)	17 489 (30.6)	6231 (31.7)	5700 (30.2)	28 236 (29.4)
Anticoagulant (injectable)	6697 (11.6)	11 182 (11.6)	5596 (9.8)	1677 (8.5)	1833 (9.7)	10 955 (11.4)
Antiplatelets	6934 (12.0)	11 593 (12.1)	5342 (9.4)	1733 (8.8)	2042 (10.8)	11 837 (12.3)
β-Blockers	41 228 (71.1)	68 498 (71.3)	38 596 (67.6)	13 263 (67.4)	13 251 (67.4)	68 782 (71.5)
Calcium channel blockers	20 300 (35.0)	33 804 (35.2)	17 373 (30.4)	6035 (30.7)	6606 (35.0)	34 232 (35.6)
Digoxin	3378 (5.8)	5686 (5.9)	4483 (7.8)	1628 (8.3)	1261 (6.7)	6079 (6.3)
Diuretics						
Loop	10 510 (18.1)	17 932 (18.7)	8968 (15.7)	2960 (15.0)	3218 (17.0)	18 424 (19.2)
Potassium sparing	4684 (8.1)	7907 (8.2)	3990 (7.0)	1323 (6.7)	1410 (7.5)	8110 (8.4)
Thiazide	11 705 (20.2)	19 516 (20.3)	5158 (9.0)	1724 (8.8)	3763 (19.9)	19 640 (20.4)
Nitrates	3103 (5.4)	5318 (5.5)	2529 (4.4)	834 (4.2)	943 (5.0)	5533 (5.8)
Statins	25 330 (43.7)	42 462 (44.2)	23 532 (41.2)	7993 (40.6)	8014 (42.4)	43 115 (44.8)
Fibrates	2218 (3.8)	3754 (3.9)	2388 (4.2)	850 (4.3)	774 (4.1)	3968 (4.1)
Amiodarone	5784 (10.0)	9740 (10.1)	5082 (8.9)	1754 (8.9)	1864 (9.9)	10 071 (10.5)
Dronedarone	1483 (2.6)	2426 (2.5)	2285 (4.0)	937 (4.8)	664 (3.5)	2933 (3.1)

Abbreviations: ACEI, angiotensin converting enzyme inhibitor; AMI, acute myocardial infarction; ARB, angiotensin receptor blocker; CHA2DS2-VASc, congestive heart failure, hypertension, age ≥75 years, diabetes, prior stroke or transient ischemic attack, or thromboembolism, vascular disease, age 65-74 years, sex category; HASBLED, hypertension, abnormal kidney and liver function, stroke, bleeding, labile international normalized ratio, elderly, drugs or alcohol; NOAC, non–vitamin K oral anticoagulant; NSAIDs, nonsteroidal anti-inflammatory drugs; SSRI, selective serotonin reuptake inhibitor.

^a^ Weighted patient characteristics tables facilitate the assessment of covariate balance after inverse probability weighting and should not be interpreted as a description of the unweighted population. Treated patients are weighted by the proportion of treated patients in the trimmed population divided by the inverse of their propensity score. Reference patients are weighted by 1 minus the proportion of treated patients in the trimmed population divided by 1 minus their propensity score.

^b^ Percentages may not total 100 due to rounding.

The number of outcome events identified and weighted incidence rates per 1000 person-years for each pairwise comparison are displayed in Table 2. The incidence rates of bleeding events were numerically higher in rivaroxaban users compared with apixaban and dabigatran users. HRs and 95% CIs for each outcome in the pairwise NOAC comparisons are shown in Table 3. Apixaban was associated with significantly lower risks of bleeding compared with rivaroxaban, while maintaining similar effectiveness in preventing thromboembolic stroke (Table 3). Rivaroxaban use was associated with increased risks of GIB (HR, 1.92; 95% CI, 1.54-2.39) and MEB (HR, 1.91; 95% CI, 1.56-2.34) but not ICH (HR, 1.63; 95% CI, 0.99-2.70), compared with apixaban. Apixaban showed superior stroke prevention compared with dabigatran (HR, 0.57; 95% CI, 0.37-0.88), with numerically lower bleeding risks. Rivaroxaban appeared to provide superior stroke protection compared with dabigatran (HR, 0.61; 95% CI, 0.39-0.94); however, a numerically increased risk of all bleeding outcomes was observed (Table 3). Results from the subgroup analyses aligned with the main analysis (eFigures 1-16 in Supplement 1).

**Table 2.** Outcome Events and Weighted Incidence Rates for Each Pairwise NOAC Comparison

Outcome	Rivaroxaban vs apixaban		Rivaroxaban vs dabigatran		Dabigatran vs apixaban	
	Rivaroxaban	Apixaban	Rivaroxaban	Dabigatran	Dabigatran	Apixaban
New users, weighted No.	57 965	96 013	57 127	19 679	18 882	96 132
Major extracranial bleed						
Outcome events, No.	224	204	188	38	39	206
Weighted incidence rate per 1000 person-years	11.6	6.4	9.9	7.3	7.8	6.4
Gastrointestinal bleed						
Outcome events, No.	191	174	157	34	37	174
Weighted incidence rate per 1000 person-years	10.0	5.4	8.3	6.6	7.2	5.4
Intracranial hemorrhage						
Outcome events, No.	34	35	29	7	8	37
Weighted incidence rate per 1000 person-years	1.8	1.1	1.5	1.4	1.7	1.2
Thromboembolic stroke						
Outcome events, No.	78	124	64	33	38	124
Weighted incidence rate per 1000 person-years	4.06	3.88	3.38	6.46	7.52	3.86

Abbreviation: NOAC, non–vitamin K oral anticoagulant.

**Table 3.** Hazard Ratios for Bleeding Events and Stroke for Each Pairwise NOAC Comparison

Outcome	Hazard ratio (95% CI)		
	Rivaroxaban vs apixaban	Rivaroxaban vs dabigatran	Dabigatran vs apixaban
Gastrointestinal bleeding	1.92 (1.54-2.39)	1.32 (0.89-1.96)	1.34 (0.88-2.05)
Major extracranial bleeding	1.91 (1.56-2.34)	1.42 (0.98-2.07)	1.22 (0.82-1.81)
Intracranial hemorrhage	1.63 (0.99-2.70)	1.18 (0.52-2.67)	1.43 (0.58-3.52)
Thromboembolic stroke	1.05 (0.77-1.44)	0.61 (0.39-0.94)	1.74 (1.13-2.68)

Abbreviation: NOAC, non–vitamin K oral anticoagulant.

The comparative risk of bleeding events and thromboembolic stroke among NOAC users younger than 65 years in this study were compared with those from Graham et al^17^ in NOAC users aged 65 years or older in Table 4. The estimates for bleeding and thromboembolic stroke largely trended in the same direction for the comparison of rivaroxaban vs apixaban. However, dabigatran use was associated with a higher thromboembolic stroke risk compared with both rivaroxaban and apixaban (rivaroxaban vs dabigatran: HR, 0.61; 95% CI, 0.39-0.94; dabigatran vs apixaban: HR, 1.74; 95% CI, 1.13-2.68) in younger patients, a finding that was not observed in older users.

**Table 4.** Comparison of Current Study Results With Findings in Older Adults

Outcome	Hazard ratio (95% CI)					
	Rivaroxaban vs apixaban		Rivaroxaban vs dabigatran		Dabigatran vs apixaban	
	Sentinel System (≤65 y)	Graham et al^17^ (≥65 y)	Sentinel System (≤65 y)	Graham et al^17^ (≥65 y)	Sentinel System (≤65 y)	Graham et al^17^ (≥65 y)
Gastrointestinal bleeding	1.92 (1.54-2.39)	2.83 (2.47-3.25)	1.32 (0.89-1.96)	1.27 (1.16-1.40)	1.34 (0.88-2.05)	2.23 (1.93-2.58)
Major extracranial bleeding	1.91 (1.56-2.34)	2.70 (2.38-3.05)	1.42 (0.98-2.07)	1.32 (1.21-1.45)	1.22 (0.82-1.81)	2.04 (1.78-2.32)
Intracranial hemorrhage	1.63 (0.99-2.70)	1.21 (0.94-1.55)	1.18 (0.52-2.67)	1.71 (1.35-2.17)	1.43 (0.58-3.52)	0.70 (0.53-0.94)
Thromboembolic stroke	1.05 (0.77-1.44)	1.02 (0.85-1.23)	0.61 (0.39-0.94)	0.90 (0.76-1.06)	1.74 (1.13-2.68)	1.14 (0.94-1.37)

## Discussion

In this large cohort study of patients with NVAF younger than 65 years, apixaban demonstrated the most favorable benefit-harm profile among the 3 NOACs studied. Apixaban was associated with significantly lower risks of MEB and GIB compared with rivaroxaban, while maintaining similar effectiveness in preventing thromboembolic stroke. Bleeding risk from rivaroxaban use remained elevated across age groups (in both this study and Graham et al^17^ in older adults) compared with apixaban. Additionally, apixaban showed superior stroke prevention compared with dabigatran, with numerically lower bleeding risks. Rivaroxaban appeared to provide superior stroke protection compared with dabigatran, suggesting a middle ground between the apixaban and dabigatran benefit-harm profiles.

The findings reveal potentially important differences in NOAC performance between younger and older users. Most notably, dabigatran use was associated with substantially higher thromboembolic stroke risk compared with both rivaroxaban and apixaban in younger patients (rivaroxaban vs dabigatran: HR, 0.61; 95% CI, 0.39-0.94; dabigatran vs apixaban: HR, 1.74; 95% CI, 1.13-2.68), a finding that was not observed in patients aged 65 years or older.^17^ This suggests that NOACs may behave differently across age groups, with dabigatran appearing less effective for stroke prevention in younger adults, although the potential for unmeasured confounding exists.

Risk of ICH also differed across age groups and comparisons. While rivaroxaban use was not associated with an increased risk of ICH compared with apixaban in younger patients in the Sentinel System (HR, 1.63; 95% CI, 0.99-2.70), there was a statistically significant increased risk of ICH observed with rivaroxaban vs dabigatran in older adults (HR, 1.71; 95% CI, 1.35-2.17) in Graham et al.^17^ This suggests that ICH risks may be more age-dependent than other bleeding complications.

The once-daily dosing of rivaroxaban, while potentially improving adherence,^33^ may contribute to higher peak anticoagulation levels early in the dosing interval, potentially explaining the observed increased bleeding risks compared with the other twice-daily dosed NOACs. Concerns about the potential for bleeding events with rivaroxaban were raised preapproval during an FDA advisory committee.^34^ While the relative risk differences between NOACs were statistically significant, it is important to note that the absolute event rates were low in this younger cohort, and the median follow-up period was relatively short (62 [IQR, 32-126] days). This shorter follow-up may be attributed to the relatively restrictive gap rule between dispensings (3 days only) applied to determine continuous use. The presentation of rates per 1000 person-years, while standard for comparative effectiveness research, may overemphasize the magnitude of absolute differences in this population with overall lower event rates.

There have been several previous observational head-to-head comparisons of NOAC safety and effectiveness.^5,6,7,8,9,10,11,12,13,14,15,16^ These studies included only limited numbers of NOAC users younger than 65 years of age, although findings largely aligned with the findings in this study despite several limitations in their approach. This was the largest study to date to compare the safety of NOACs with each other in users younger than 65 years, with approximately 96 000 apixaban users, 58 000 rivaroxaban users, and 20 000 dabigatran users included in the analyses. Despite the large numbers of NOAC users, average treatment effect weights were used for balancing characteristics in pairwise comparisons to preserve the sample size. A range of relevant clinical outcomes were evaluated facilitating assessment of the overall balance of NOAC benefits and harms and the robustness of findings were tested in multiple subgroups.

Atrial fibrillation is associated with substantial long-term morbidity and mortality following ischemic stroke. Recent observational data^35^ suggest that patients with atrial fibrillation experience higher early poststroke mortality and worse overall prognosis, emphasizing the importance of effective stroke prevention strategies and optimizing anticoagulation. At the same time, evidence from clinical practice^36^ indicates that anticoagulation in patients perceived to be at high bleeding risk remains suboptimal, with frequent nonuse or inappropriate dose reduction of NOACs, contrary to guideline recommendations. Our findings that apixaban demonstrated a more favorable benefit-harm profile among younger patients with NVAF may help support careful anticoagulant selection to optimize stroke prevention while minimizing bleeding risk.

### Limitations

Several limitations inherent to the observational study design should be considered when interpreting these findings. First, although we used a new-user active-comparator design with extensive PS adjustment, residual confounding remains a possibility. In particular, confounding by indication and treatment channeling may persist if clinicians preferentially prescribed specific NOACs based on unmeasured factors such as frailty, perceived bleeding risk, or patient preference. To the extent that apixaban may have been preferentially prescribed to patients perceived to be at higher bleeding risk, such confounding would be expected to inflate the risk of bleeding and attenuate toward the null or potential harm any true protective effect.

Second, outcome misclassification is possible in administrative claims data when using diagnosis codes rather than adjudicated events. However, we used validated algorithms with high PPVs for stroke and bleeding outcomes and any misclassification was likely nondifferential and would be expected to bias results toward the null. Third, claims data lack detailed clinical and lifestyle information, and data on over-the-counter medication use such as aspirin, which may influence both treatment selection and outcomes. While inverse probability of treatment weighting achieved good balance across measured covariates, unmeasured confounding cannot be excluded. Also, follow-up was censored at treatment discontinuation or switching, which may introduce informative censoring if these events were related to early symptoms or evolving risk. However, this approach reflects an as-treated approach to follow-up that aligns with our clinical question of comparative risks during periods of active NOAC use. Only initiators of standard-dose NOACs were included in this study, and it is not clear whether the effects vary in patients treated with lower doses. Similarly, only first-time users of NOACs for stroke prevention in NVAF were included, and results might differ in patients switching to NOACs from warfarin. As this was an observational study, confounding by factors were not adjusted for in the analysis or by residual channeling bias and may have influenced the results. Given the study period spanned from 2010 to 2022, prescribing patterns, reimbursement policies, and clinical guidelines for NOAC use could have changed. Differences in timing of market entry, evolving clinical experience, and guideline recommendations may have influenced clinician choice of NOAC over time. Although calendar year was incorporated into the PS models, residual confounding related to time-varying treatment selection cannot be fully excluded. However, the observed associations were generally consistent across subgroups and aligned with findings from prior studies conducted in different calendar periods, suggesting that the main conclusions are unlikely to be driven solely by temporal confounding. Mortality is not reliably captured from the commercial claims insurance data partners within the Sentinel System; therefore, we were unable to assess mortality as an outcome in our study and were unable to conduct a competing risk analysis for mortality. However, all-cause mortality is expected to be substantially lower in patients younger than 65 years compared with older NOAC users. The HRs computed were comparison-specific and should not be interpreted as transitive across different pairwise analyses. An additional limitation is the relatively short duration of follow-up and the low number of outcome events in this younger population. Studies with longer follow-up would help clarify whether the observed relative differences persist or widen over extended treatment durations.

## Conclusions

In this observational cohort study of patients younger than 65 years of age treated with NOACs for NVAF, apixaban was associated with the most favorable benefit-harm profile. While the results suggest that rivaroxaban may have provided greater thromboembolic stroke prevention than dabigatran, it was associated with higher bleeding risks than apixaban without additional stroke prevention. The higher stroke risks observed with dabigatran in younger patients suggest important age-related differences in effectiveness that warrant further investigation.

## Notes

Supplement. **eTable 1.** Adjusted Hazard Ratios Comparing Rivaroxaban vs Dabigatran in those 65 years and older in the study by Graham et al, and an analysis in the Sentinel system using the Medicare data partner only **eTable 2.** Adjusted Hazard Ratios Comparing Rivaroxaban vs Apixaban in those 65 years and older in the study by Graham et al, and an analysis in the Sentinel system using the Medicare data partner only **eTable 3.** Adjusted Hazard Ratios Comparing Dabigatran vs Apixaban in those 65 years and older in the study by Graham et al, and an analysis in the Sentinel system using the Medicare data partner only **eTable 4.** Aggregated weighted complete list of patient characteristics of NOAC users in the Sentinel Distributed Database from October 19, 2010 to February 28, 2022* **eTable 5** Aggregated unadjusted characteristics of rivaroxaban and apixaban users in the Sentinel Distributed Database from October 19, 2010, to February 28, 2022 **eTable 6.** Aggregated unadjusted characteristics of rivaroxaban and dabigatran users in the Sentinel Distributed Database from October 19, 2010, to February 28, 2022 **eTable 7.** Aggregated unadjusted characteristics of dabigatran and apixaban users in the Sentinel Distributed Database from October 19, 2010, to February 28, 2022 **eFigure 1.** Adjusted hazard ratios for thromboembolic stroke comparing rivaroxaban and apixaban users, overall and by subgroup **eFigure 2.** Adjusted hazard ratios for major extracranial bleeding comparing rivaroxaban and apixaban users, overall and by subgroup **eFigure 3.** Adjusted hazard ratios for gastrointestinal hemorrhage comparing rivaroxaban and apixaban users, overall and by subgroup **eFigure 4.** Adjusted hazard ratios for intracranial hemorrhage comparing rivaroxaban and apixaban users, overall and by subgroup **eFigure 5.** Adjusted hazard ratios for thromboembolic stroke comparing rivaroxaban and dabigatran users, overall and by subgroup **eFigure 6.** Adjusted hazard ratios for major extracranial bleeding comparing rivaroxaban and dabigatran users, overall and by subgroup **eFigure 7.** Adjusted hazard ratios for gastrointestinal hemorrhage comparing rivaroxaban and dabigatran users, overall and by subgroup **eFigure 8.** Adjusted hazard ratios for intracranial hemorrhage comparing rivaroxaban and dabigatran users, overall and by subgroup **eFigure 9.** Adjusted hazard ratios for thromboembolic stroke comparing dabigatran and apixaban users, overall and by age and sex subgroups **eFigure 10.** Adjusted hazard ratios for thromboembolic stroke comparing dabigatran and apixaban users, CHADS2-VASc and HASBLED subgroups **eFigure 11.** Adjusted hazard ratios for major extracranial bleeding comparing dabigatran and apixaban users, overall and by age and sex subgroups **eFigure 12.** Adjusted hazard ratios for major extracranial bleeding comparing dabigatran and apixaban users, CHADS2-VASc and HASBLED subgroups **eFigure 13.** Adjusted hazard ratios for gastrointestinal hemorrhage comparing dabigatran and apixaban users, overall and by subgroup **eFigure 14.** Adjusted hazard ratios for gastrointestinal hemorrhage comparing dabigatran and apixaban users, by CHADS2-VASc and HASBLED subgroups **eFigure 15.** Adjusted hazard ratios for intracranial hemorrhage comparing dabigatran and apixaban users, overall and by subgroup **eFigure 16.** Adjusted hazard ratios for intracranial hemorrhage comparing dabigatran and apixaban users, CHADS2-VASc and HASBLED subgroups

Supplement 2.
Data Sharing Statement

## References

[1] Connolly SJ, Ezekowitz MD, Yusuf S, ; RE-LY Steering Committee and Investigators. Dabigatran versus warfarin in patients with atrial fibrillation. N Engl J Med. 2009;361(12):1139-1151. doi:10.1056/NEJMoa0905561 19717844

[2] Giugliano RP, Ruff CT, Braunwald E, ; ENGAGE AF-TIMI 48 Investigators. Edoxaban versus warfarin in patients with atrial fibrillation. N Engl J Med. 2013;369(22):2093-2104. doi:10.1056/NEJMoa1310907 24251359

[3] Granger CB, Alexander JH, McMurray JJ, ; ARISTOTLE Committees and Investigators. Apixaban versus warfarin in patients with atrial fibrillation. N Engl J Med. 2011;365(11):981-992. doi:10.1056/NEJMoa1107039 21870978

[4] Patel MR, Mahaffey KW, Garg J, ; ROCKET AF Investigators. Rivaroxaban versus warfarin in nonvalvular atrial fibrillation. N Engl J Med. 2011;365(10):883-891. doi:10.1056/NEJMoa1009638 21830957

[5] Abraham NS, Noseworthy PA, Yao X, Sangaralingham LR, Shah ND. Gastrointestinal safety of direct oral anticoagulants: a large population-based study. Gastroenterology. 2017;152(5):1014-1022.e1. doi:10.1053/j.gastro.2016.12.018 28043907

[6] Chan YH, Kuo CT, Yeh YH, . Thromboembolic, bleeding, and mortality risks of rivaroxaban and dabigatran in Asians with nonvalvular atrial fibrillation. J Am Coll Cardiol. 2016;68(13):1389-1401. doi:10.1016/j.jacc.2016.06.062 27659460

[7] Gorst-Rasmussen A, Lip GY, Bjerregaard Larsen T. Rivaroxaban versus warfarin and dabigatran in atrial fibrillation: comparative effectiveness and safety in Danish routine care. Pharmacoepidemiol Drug Saf. 2016;25(11):1236-1244. doi:10.1002/pds.4034 27229855

[8] Graham DJ, Reichman ME, Wernecke M, . Stroke, bleeding, and mortality risks in elderly Medicare beneficiaries treated with dabigatran or rivaroxaban for nonvalvular atrial fibrillation. JAMA Intern Med. 2016;176(11):1662-1671. doi:10.1001/jamainternmed.2016.5954 27695821

[9] Grymonprez M, De Backer TL, Bertels X, Steurbaut S, Lahousse L. Long-term comparative effectiveness and safety of dabigatran, rivaroxaban, apixaban and edoxaban in patients with atrial fibrillation: a nationwide cohort study. Front Pharmacol. 2023;14:1125576. doi:10.3389/fphar.2023.1125576 36817122 PMC9932194

[10] Hernandez I, Zhang Y. Comparing stroke and bleeding with rivaroxaban and dabigatran in atrial fibrillation: analysis of the US Medicare Part D data. Am J Cardiovasc Drugs. 2017;17(1):37-47. doi:10.1007/s40256-016-0189-9 27637493 PMC6572759

[11] Hernandez I, Zhang Y, Saba S. Comparison of the effectiveness and safety of apixaban, dabigatran, rivaroxaban, and warfarin in newly diagnosed atrial fibrillation. Am J Cardiol. 2017;120(10):1813-1819. doi:10.1016/j.amjcard.2017.07.092 28864318

[12] Lip GY, Pan X, Kamble S, . Major bleeding risk among non-valvular atrial fibrillation patients initiated on apixaban, dabigatran, rivaroxaban or warfarin: a “real-world” observational study in the United States. Int J Clin Pract. 2016;70(9):752-763. doi:10.1111/ijcp.12863 27550177 PMC5129572

[13] Lip GYH, Keshishian AV, Zhang Y, . Oral anticoagulants for nonvalvular atrial fibrillation in patients with high risk of gastrointestinal bleeding. JAMA Netw Open. 2021;4(8):e2120064. doi:10.1001/jamanetworkopen.2021.20064 34398204 PMC8369361

[14] Noseworthy PA, Yao X, Abraham NS, Sangaralingham LR, McBane RD, Shah ND. Direct comparison of dabigatran, rivaroxaban, and apixaban for effectiveness and safety in nonvalvular atrial fibrillation. Chest. 2016;150(6):1302-1312. doi:10.1016/j.chest.2016.07.013 27938741

[15] Palamaner Subash Shantha G, Bhave PD, Girotra S, . Sex-specific comparative effectiveness of oral anticoagulants in elderly patients with newly diagnosed atrial fibrillation. Circ Cardiovasc Qual Outcomes. 2017;10(4):e003418. doi:10.1161/CIRCOUTCOMES.116.003418 28408716 PMC5412710

[16] Sherid M, Sifuentes H, Sulaiman S, . Risk of gastrointestinal bleeding with dabigatran: a head-to-head comparative study with rivaroxaban. Digestion. 2014;90(2):137-146. doi:10.1159/000365967 25278002

[17] Graham DJ, Baro E, Zhang R, . Comparative stroke, bleeding, and mortality risks in older Medicare patients treated with oral anticoagulants for nonvalvular atrial fibrillation. Am J Med. 2019;132(5):596-604.e11. doi:10.1016/j.amjmed.2018.12.023 30639551

[18] Maro JC, Nguyen MD, Kolonoski J, . Six years of the US Food and Drug Administration’s postmarket active risk identification and analysis system in the sentinel initiative: implications for real world evidence generation. Clin Pharmacol Ther. 2023;114(4):815-824. doi:10.1002/cpt.2979 37391385

[19] Rosati K, Jorgensen N, Soliz M, Evans BJ. Sentinel initiative principles and policies HIPAA and common rule compliance in the sentinel initiative. US Food and Drug Administration. February 1, 2018. Accessed March 4, 2026. https://www.sentinelinitiative.org/sites/default/files/communications/publications-presentations/HIPAA-Common-Rule-Compliance-in-Sentinel-Initiative.pdf

[20] Office for Human Research Protections. 45 CFR §46.102(l)(2). US Department of Health and Human Services. Accessed September 30, 2024. https://www.hhs.gov/ohrp/regulations-and-policy/regulations/45-cfr-46/index.html

[21] Kokotailo RA, Hill MD. Coding of stroke and stroke risk factors using *International Classification of Diseases*, revisions 9 and 10. Stroke. 2005;36(8):1776-1781. doi:10.1161/01.STR.0000174293.17959.a1 16020772

[22] Roumie CL, Mitchel E, Gideon PS, Varas-Lorenzo C, Castellsague J, Griffin MR. Validation of ICD-9 codes with a high positive predictive value for incident strokes resulting in hospitalization using Medicaid health data. Pharmacoepidemiol Drug Saf. 2008;17(1):20-26. doi:10.1002/pds.1518 17979142

[23] Tirschwell DL, Longstreth WT Jr. Validating administrative data in stroke research. Stroke. 2002;33(10):2465-2470. doi:10.1161/01.STR.0000032240.28636.BD 12364739

[24] Cunningham A, Stein CM, Chung CP, Daugherty JR, Smalley WE, Ray WA. An automated database case definition for serious bleeding related to oral anticoagulant use. Pharmacoepidemiol Drug Saf. 2011;20(6):560-566. doi:10.1002/pds.2109 21387461 PMC3365595

[25] Panozzo CA, Welch EC, Woodworth TS, . Assessing the impact of the new ICD-10-CM coding system on pharmacoepidemiologic studies-an application to the known association between angiotensin-converting enzyme inhibitors and angioedema. Pharmacoepidemiol Drug Saf. 2018;27(8):829-838. doi:10.1002/pds.4550 29947045

[26] Panozzo CA, Woodworth TS, Welch EC, . Early impact of the ICD-10-CM transition on selected health outcomes in 13 electronic health care databases in the United States. Pharmacoepidemiol Drug Saf. 2018;27(8):839-847. doi:10.1002/pds.4563 29947033

[27] Centers for Medicare & Medicaid Services. ICD-10 Files & News Archive. Accessed September 30, 2024. https://www.cms.gov/medicare/coding-billing/icd-10-codes/icd-10-cm-icd-10-pcs-gem-archive

[28] Lip GY, Nieuwlaat R, Pisters R, Lane DA, Crijns HJ. Refining clinical risk stratification for predicting stroke and thromboembolism in atrial fibrillation using a novel risk factor-based approach: the Euro Heart Survey on atrial fibrillation. Chest. 2010;137(2):263-272. doi:10.1378/chest.09-1584 19762550

[29] Pisters R, Lane DA, Nieuwlaat R, de Vos CB, Crijns HJ, Lip GY. A novel user-friendly score (HAS-BLED) to assess 1-year risk of major bleeding in patients with atrial fibrillation: the Euro Heart Survey. Chest. 2010;138(5):1093-1100. doi:10.1378/chest.10-0134 20299623

[30] Desai RJ, Franklin JM. Alternative approaches for confounding adjustment in observational studies using weighting based on the propensity score: a primer for practitioners. BMJ. 2019;367:l5657. doi:10.1136/bmj.l5657 31645336

[31] Cole SR, Hernán MA. Constructing inverse probability weights for marginal structural models. Am J Epidemiol. 2008;168(6):656-664. doi:10.1093/aje/kwn164 18682488 PMC2732954

[32] Shu D, Yoshida K, Fireman BH, Toh S. Inverse probability weighted Cox model in multi-site studies without sharing individual-level data. Stat Methods Med Res. 2020;29(6):1668-1681. doi:10.1177/0962280219869742 31448681 PMC7042068

[33] Kubitza D, Berkowitz SD, Misselwitz F. Evidence-based development and rationale for once-daily rivaroxaban dosing regimens across multiple indications. Clin Appl Thromb Hemost. 2016;22(5):412-422. doi:10.1177/1076029616631427 26893445 PMC4888194

[34] US Food and Drug Administration. 2011 Meeting materials, Cardiovascular and Renal Drugs Advisory Committee. Accessed September 19, 2024. https://wayback.archive-it.org/7993/20170403223812/https://www.fda.gov/AdvisoryCommittees/CommitteesMeetingMaterials/Drugs/CardiovascularandRenalDrugsAdvisoryCommittee/ucm250287.htm

[35] Tracz J, Gorczyca-Głowacka I, Rosołowska A, Wożakowska-Kapłon B. Long-term outcomes after stroke in patients with atrial fibrillation: a single center study. Int J Environ Res Public Health. 2023;20(4):3491. doi:10.3390/ijerph20043491 36834183 PMC9967874

[36] Maciorowska M, Uziębło-Życzkowska B, Gorczyca-Głowacka I, . Oral anticoagulation therapy in atrial fibrillation patients at high risk of bleeding: clinical characteristics and treatment strategies based on data from the Polish multicenter register of atrial fibrillation (POL-AF). Kardiol Pol. 2024;82(1):37-45. doi:10.33963/v.kp.98356 38230462

