# Supplementary material for: Stroke and Bleeding Risks With Non–Vitamin K Oral Anticoagulants in Nonvalvular Atrial Fibrillation
Source: JAMA Netw Open. 2026 Apr 24;9(4):e269082. doi: 10.1001/jamanetworkopen.2026.9082 (PMC13109794; doi:10.1001/jamanetworkopen.2026.9082)
Supplement: Supplement 2. — Data Sharing Statement [file jamanetwopen-e269082-s002.pdf]

Data Sharing Statement

Bradley. Stroke and Bleeding Risks With Non–Vitamin K Oral Anticoagulants in Nonvalvular Atrial Fibrillation. *JAMA Netw Open*. Published April 24, 2026.
doi:10.1001/jamanetworkopen.2026.9082

Data
Data available: No

Additional Information
Explanation for why data not available: FDA Sentinel System Data is not available for sharing
